# Accuracy of comparison decisions by forensic firearms examiners

**DOI:** 10.1111/1556-4029.15152

**Published:** 2022-10-01

**Authors:** Keith L. Monson, Erich D. Smith, Eugene M. Peters

**Affiliations:** ^1^ Federal Bureau of Investigation Laboratory Quantico Virginia USA

**Keywords:** accuracy, black box, consecutive manufacture, decision analysis, error rate, firearms and toolmark identification, foundational validity, open set design, reliability, subclass

## Abstract

This black box study assessed the performance of forensic firearms examiners in the United States. It involved three different types of firearms and 173 volunteers who performed a total of 8640 comparisons of both bullets and cartridge cases. The overall false‐positive error rate was estimated as 0.656% and 0.933% for bullets and cartridge cases, respectively, while the rate of false negatives was estimated as 2.87% and 1.87% for bullets and cartridge cases, respectively. The majority of errors were made by a limited number of examiners. Because chi‐square tests of independence strongly suggest that error probabilities are not the same for each examiner, these are maximum‐likelihood estimates based on the beta‐binomial probability model and do not depend on an assumption of equal examiner‐specific error rates. Corresponding 95% confidence intervals are (0.305%, 1.42%) and (0.548%, 1.57%) for false positives for bullets and cartridge cases, respectively, and (1.89%, 4.26%) and (1.16%, 2.99%) for false negatives for bullets and cartridge cases, respectively. The results of this study are consistent with prior studies, despite its comprehensive design and challenging specimens.

**Highlights**

- A total of 173 qualified forensic firearms examiners made 8640 comparisons.
- False‐positive error rate: 0.656% (bullets) and 0.933% (cartridge cases).
- False‐negative error rate: 2.87% (bullets) and 1.87% (cartridge cases).
- Each examiner has an individual error probability.
- The majority of errors were made by a limited number of examiners.
- Inconclusive decisions arose more frequently with comparisons of nonmatching sets.

## 1 INTRODUCTION

Forensic firearms examination, like other pattern evidence analysis disciplines (e.g., latent fingerprints and LFP), relies on expertise, training, and judgment to make comparisons between questioned, evidentiary specimens and known exemplars for source attribution decisions. The Federal Bureau of Investigation (FBI) Laboratory initiated research to strengthen the admissibility of pattern evidence examination decisions in 2006, starting with fingerprint comparisons, and publishing the first results in 2011 [1]. In 2009, a committee convened by the National Research Council (NRC) offered recommendations for improvements to forensic science practice [2]. Among these, Recommendation 3 emphasized the need for more studies to establish the scientific bases that demonstrate the validity, reliability, and accuracy of forensic methods.

The President's Council of Advisors on Science and Technology (PCAST) published a 2016 report that reviewed the scientific validity of a number of feature comparison analysis methods, including LFP and firearms [3]. PCAST reviewed a Department of Energy report in the public domain [4] in the area of firearm examinations. Concerning fingerprints, PCAST concluded that the design of and results from an LFP study [1] were instrumental in establishing the validity of LFP comparisons. The experimental methodology used in both these studies, particularly their open set designs, was described by PCAST to be of high quality. Nevertheless, PCAST, like the NRC report before it, recommended additional research. For the discipline of firearms analysis, Finding 6 prescribed additional well‐designed black box studies to determine error rates, establish foundational validity, and support testimony.

Previous studies by forensic firearms examiners and independent researchers have examined the accuracy of firearm examiner decisions [5, 6, 7, 8, 9]. Since the publication of the PCAST report, a number of firearms examiners and independent researchers have conducted additional investigations dealing with various aspects of comparative examinations. These include the estimation of examiner error rates [10, 11, 12, 13, 14, 15, 16, 17, 18], statistical evaluation methods in the Identification of toolmarks [19, 20], and efforts to produce either automated or computer‐based objective determinations [15, 21, 22, 23, 24, 25, 26, 27, 28, 29, 30, 31]. Additionally, several compilations contain general discussions and document research efforts as applied to firearms and toolmark examinations [32, 33, 34, 35, 36, 37].

This study reports results related to examiner accuracy, the ability of an examiner to correctly identify a known match or to eliminate a known nonmatch (error rate). It is part of a larger study that included intra‐examiner repeatability and inter‐examiner reproducibility of examination results and also examined effects related to firearm make, tool wear (related to manufacturing or firing order), and human factors (e.g., years of experience and perceived difficulty). Complementary papers will detail the latter aspects.

The basic task of the study was the comparison of unknown cartridge cases and bullet specimens by a firearms examiner who volunteered to participate. Fired bullets and cartridge cases were obtained using three different manufacturers of firearms and a single brand of ammunition. The firearms and ammunition selected for this study were selected for their propensity to produce challenging and ambiguous test specimens, creating difficult comparisons for examiners. Firearms were chosen whose design precluded the creation of aperture drag (which is readily identifiable) and were likely to be highly similar and to display subclass characteristics (having been collected after consecutive or sequential manufacture and incorporating a variable range of firing intervals between the known and questioned specimens in each set). The ammunition used had steel cartridge cases and steel‐jacketed bullets (steel, being harder than brass, is less likely to be marked) [33]. Thus, the study was designed to be a rigorous trial of examiner ability; as a result, error rates derived from this study may provide an upper bound to a possible error in operational casework, as evidentiary specimens may generally be assumed to be less challenging than those used in this study. Although generally analogous to the previous Ulery et al. LFP [1] and Baldwin et al.'s firearms [4] studies in terms of experimental design and methodology, this study was considerably broader than Baldwin in that it involved both cartridge cases and bullets and took into account additional parameters that might affect examiner accuracies such as challenging comparisons, manufacturing conditions, presence of subclass characteristics, and firing order separation. An open set design, where there may not necessarily be a match for every questioned specimen, was implemented. An open set design avoids the underestimation of false positives inherent in a closed set but may increase the number of Inconclusive decisions.

## 2 MATERIALS AND METHODS

Full details of the planning, design, and logistics of the study, including the rationale for choices of specific firearms and ammunition is provided by Monson, et al. [33]. This study was designed as a declared double‐blind “black box” investigation, in which the examiners were aware of their participation in a study. Contact between the participating examiner subjects and the experimental team was precluded, both to preserve the anonymity of the participants and prevent any interactions between participants and investigators that might result in bias [33, 38, 39, 40, 41]. Duties related to communication with the participants and generation and scoring of the specimens provided for examination were strictly compartmentalized. No specimen‐specific information was shared between the compartmentalized communication group and the experimental/analysis group. The study was reviewed and approved by the cognizant Institutional Review Board (IRB) and all results were kept anonymous, pursuant to IRB requirements. Given a large number of organizations represented and the number of specimens to be compared over multiple rounds of submission, conducting a fully double‐blind study (where the participants were unaware that they were participating in a research study and neither they nor the study administrators knew the correct answers) would have presented nearly insurmountable logistical challenges, potentially compromising the anonymity of participants, and creating the risk of co‐mingling experimental samples with real casework evidentiary materials. Moreover, conducting a fully double‐blind study was precluded by the statutory and IRB requirements to obtain informed consent from the participating examiners [42, 43].

Broad calls for volunteers were made through the Association of Firearm and Toolmark Examiners (AFTE) website; by announcements and presentations at national forensic meetings including AFTE, Association of Crime Laboratory Directors (ASCLD), and National Institute of Science and Technology (NIST); through e‐mail lists maintained by AFTE (AFTE membership was not required for participation); and through national/international listservs. Due to difficulties with mailing bullets and cartridge cases overseas, a decision was made to accept only examiners within the United States. Examiners associated with the FBI were excluded to eliminate possible conflicts of interest. Initially, 256 examiners expressed interest, but only those who were willing and able to commit to the substantial effort that was required persisted. This was a self‐selection process over which we had no control. A total of 173 qualified examiners working in 41 states were active participants in the accuracy study. Of the 157 participants who indicated whether their laboratory was accredited, 18 responded negatively. Median examiner experience was 9 years [33].

The ammunition used was Wolf Polyformance 9 mm Luger (9 × 19 mm). These cartridges are polymer coated, having steel cartridge cases with brass primers and 115‐grain, copper‐coated, steel‐jacketed, and lead bullets [44]. Fired cartridge cases were collected from 10 Jimenez JA‐Nine and 27 Beretta M9A3‐FDE semiautomatic pistols. Fired bullets were collected from 11 Ruger SR‐9c and the same 27 Beretta semiautomatic pistols. The number of specimens collected for this study and use in other aspects of the research program was 700 specimens per Beretta firearm and 850 specimens per non‐Beretta firearm, for both cartridge cases and bullets, from 28,250 test fires. The majority of the firearms had newly and consecutively or sequentially manufactured barrels and slides. Four used Beretta firearms, chosen at random from those retained from adjudicated cases and therefore of unknown history, served as ground truth nonmatch firearms. Each new firearm was test fired before specimen collection began (30 times for Jimenez and 60 times for the others). The break‐in firings were employed to stabilize internal wear within the firearms and achieve consistent and reproducible toolmarks [45]. All firearms were cleaned with a dry linen patch after firing every 250 cartridges during the collection process.

Test packets and comparison sets were assembled using the following parameters:

1. An open set design was used, i.e., there was no match for every questioned specimen.
2. Only cartridge cases and bullets fired from the same make and model firearm were in each comparison set.
3. Each comparison set, consisting of one questioned item and two reference items, represented an independent comparison, unrelated to any other set in the test packet.
4. The overall proportion of known (true) matches in the test packets averaged 33% but varied from 20% to 46% between bullets and cartridge cases within a test packet and across all test packets.
5. The ratio of non‐Beretta‐to‐Beretta specimens (for cartridge cases and bullets) in a test packet was 1:2.

Each test packet mailed to examiners consisted of 30 comparison specimen sets, with 15 cartridge case sets and 15 bullet sets. Each comparison set consisted of a single questioned specimen to be compared to two known specimens, the latter fired within the same sequence group of 50 and from the same firearm. The cartridge case comparisons were 5 sets of Jimenez and 10 sets of Beretta specimens, and the bullet comparisons were 5 sets of Ruger and 10 sets of Beretta specimens. Bar code labeling, distribution, and tracking of specimens are described in Supplement S1. The firing order was not disclosed to participating subjects but was tracked to evaluate any effect of firearm wear on examiners' analysis results. Results related to firearm manufacture and wear will be discussed in another paper.

Specimens were provided to the volunteers through a series of mailings. Via an instruction sheet [33], participants were specifically asked not to use their laboratory or agency quality assurance processes and not to discuss their conclusions with others. Following examination using a comparison microscope, examiners were asked to render a decision for each individual comparison set analyzed as *Identification*, *Elimination*, *Inconclusive (A, B, or C),* or *Unsuitable* using the AFTE range of conclusions shown in Table 1 [46]. They retained the prerogative not to declare exclusions based on individual characteristics if that was their laboratory's policy, which applied to 7% of the examiners.

**TABLE 1:** AFTE range of conclusions [46]

Identification Agreement of a combination of individual characteristics and all discernible class characteristics where the extent of the agreement exceeds that which can occur in the comparison of toolmarks made by different tools and is consistent with the agreement demonstrated by toolmarks known to have been produced by the same tool. Inconclusive Some agreement of individual characteristics and all discernible class characteristics, but insufficient for an identification. Agreement of all discernible class characteristics without agreement or disagreement of individual characteristics due to an absence, insufficiency, or lack of reproducibility. Agreement of all discernible class characteristics and disagreement of individual characteristics, but insufficient for an elimination. Elimination The significant disagreement of discernible class characteristics and/or individual characteristics. Unsuitable for examination.

The 173 participating firearm examiners provided comparison results for a total of 668 test packets, resulting in 8640 comparisons of fired cartridge cases and bullets. If a decision error was noted, the comparison set was barcode read, and the information was compared to ground truth to verify the error.

## 3 RESULTS

A summary of the evaluations for each of the 4320 bullets and 4320 cartridge case comparisons used to determine accuracy, by reference to the ground truth status of each comparison set, is given in Table 2. The heading “ID” indicates an examiner made an Identification decision; and Inconcl‐A, B, and C decisions correspond to the Inconclusive determinations defined by the AFTE range of conclusions (Table 1). The final column labeled “Other” in Table 2 includes unrecorded conclusions (6 bullet sets and 7 cartridge case sets), those recorded as Inconclusive without a level designation (A, B, or C; occurred for 1 cartridge case set), or where multiple Inconclusive levels were recorded (9 bullet sets and 3 cartridge case sets).

**TABLE 2:** Bullet and cartridge case comparison counts relative to ground truth (errors highlighted in bold)

	ID	Examiner Conclusion					
		Inconcl‐A	Inconcl‐B	Inconcl‐C	Elimination	Unsuitable	Other
Bullet evaluations relative to ground truth							
Matching	1076	127	125	36	**41**	22	2
Nonmatching	**20**	268	848	745	961	36	13
Cartridge case evaluations relative to ground truth							
Matching	1056	177	140	22	**25**	23	2
Nonmatching	**26**	177	637	620	1375	32	9

Throughout this study, an error is defined as an instance in which Elimination was declared for a true matching set, or Identification was declared for a true nonmatching set. The counts of such errors are highlighted in bold in Table 2. Counts recorded as unsuitable or in the other category are not included in accuracy calculations, as no comparison was performed. Summary conclusion percentages are computed by dividing each of the entries in Table 2 by its corresponding row sum and are presented in Table 3. For example, the proportion of false positives (False‐Pos) equals the total number of incorrect Identification conclusions over the total number of conclusions:

**TABLE 3:** Bullet and cartridge case comparison percentages relative to ground truth (errors highlighted in bold)

	ID	Examiner Conclusion				
		Inconclusive‐A	Inconclusive‐B	Inconclusive‐C	Elimination	Total Sets
Bullet evaluations relative to ground truth						
Matching	76.60%	9.04%	8.90%	2.56%	**2.92%**	1405
Nonmatching	**0.70%**	9.43%	29.80%	26.20%	33.80%	2842
Cartridge case evaluations relative to ground truth						
Matching	74.37%	12.46%	9.86%	1.55%	**1.76%**	1420
Nonmatching	**0.92%**	6.24%	22.47%	21.87%	48.50%	2835

(1) False−Pos=100%*IDID+Inconcl‐A+Inconcl‐B+Inconcl‐C+Elimination=100%*2020+268+848+745+961=0.704%

and the proportion of Elimination conclusions among matching bullet sets (or false negatives, False‐Neg) is

(2) False−Neg=100%*EliminationID+Inconcl‐A+Inconcl‐B+Inconcl‐C+Elimination=100%*411076+127+848+125+36+41=2.92%

after the removal of the comparisons represented in the Unsuitable and Other columns of Table 2.

The numbers of examiners making each type of error are shown in Table 4. Error prevalence showed no correlation with the type of training received or years of professional experience (data not shown). The false‐positive and false‐negative errors were made by a relatively small subset of the examiners, as was reported previously [4]. No errors were made by 139 of 173 examiners (80%), either false positive or false negative when examining bullets; 137 examiners made no errors of either kind when examining cartridge cases (79%). Errors were made by 34 of the 173 examiners when examining bullets (20%) and 36 of 173 examiners for cartridge cases (21%). Six participants made errors with both specimen types.

**TABLE 4:** Number of examiners making no errors, false‐positive errors, false‐negative errors, or a combination of both

	False negatives					Total examiners
Bullet evaluations						
False positives	0	1	2	3	4	
0	139	17	5	1	1	163
1	3	1			1	5
2	2					2
3	1					1
4	1		1			2
Total examiners	146	18	6	1	2	173
	False negatives					Total examiners
Cartridge case evaluations						
False positives	0	1	2	3	4	
0	137	14	4			155
1	9	3				12
2	4					4
3	2					2
4						0
Total examiners	152	17	4	0	0	173

Examiners showed marked variability in their frequency of making definitive conclusions. This is illustrated in Figure 2 for comparisons of known matching and nonmatching bullets and cartridge cases. Figure 2 shows the percentage of completed comparisons (which varies by the examiner) in which each AFTE decision category was invoked (ordinate) by each of the 173 examiners (abscissa). In charts of matching comparisons, the data were sorted by Identifications in descending order. Nonmatching comparisons were sorted by Eliminations in descending order. Correct definitive conclusions are in green (i.e., Identification of known matches and Elimination of known nonmatches), while errors are in red (incorrect Identification of known nonmatches and Elimination of known matches). In all charts, levels of Inconclusive are coded: Inc‐A in pale green, Inc‐B in yellow, and Inc‐C in amber. It is emphasized that, although the comparison sets were similar in that they were derived from the same ammunition and group of firearms, every comparison set was different, and the number of comparisons completed by each examiner varied. The number of comparison sets reported by different examiners varied from 2 to 17 (matching sets) and from 7 to 28 (nonmatching sets). As also observed among LFP examiners [47], differences in the observed rate of definitive conclusions are due to a combination of examiner skill, risk tolerance, and the number of, and challenges presented by, the particular comparison sets each examiner received.

**FIGURE 1:**
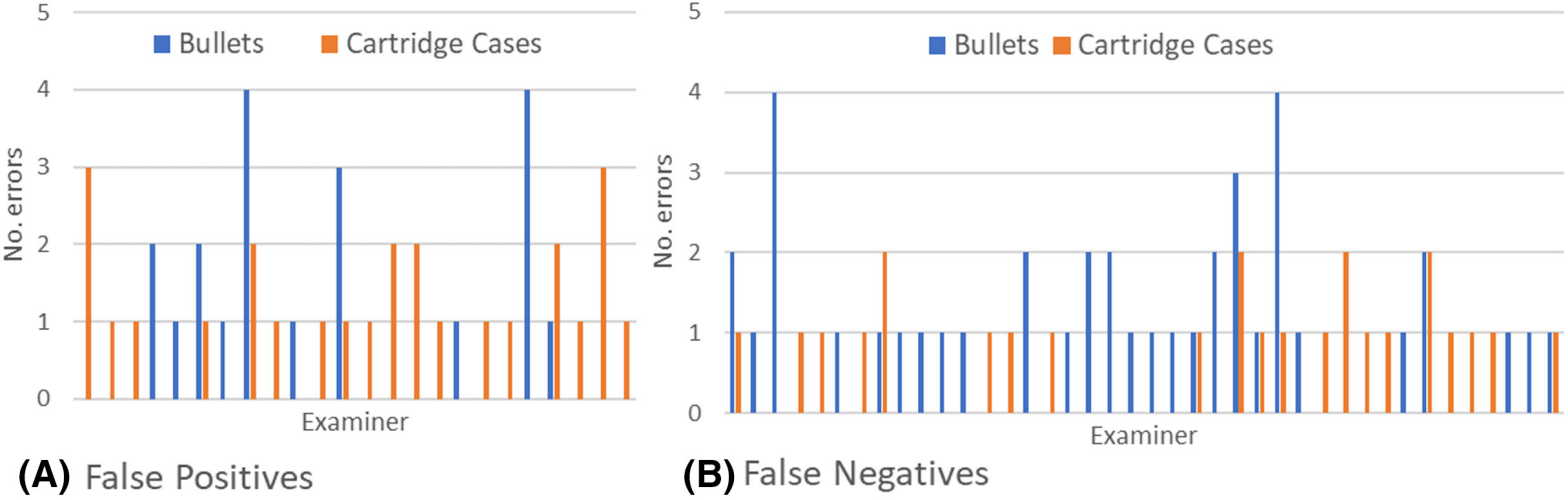
Erroneous conclusions by examiners: (A) false positives (total of 20 bullet and 26 cartridge case nonmatching sets) and (B) false negatives (total of 41 bullet and 25 cartridge case matching sets). [Color figure can be viewed at wileyonlinelibrary.com]

For known matches, the overall trend in Identification and Inconclusive decision frequency is similar for bullet and cartridge case sets, with the latter showing more examiners making Identifications. As in Table 4 and Figure 1, comparisons of nonmatching bullet sets resulted in fewer eliminations, consequently with more Inconclusive decisions, than seen in comparisons of nonmatching cartridge case sets.

**FIGURE 2:**
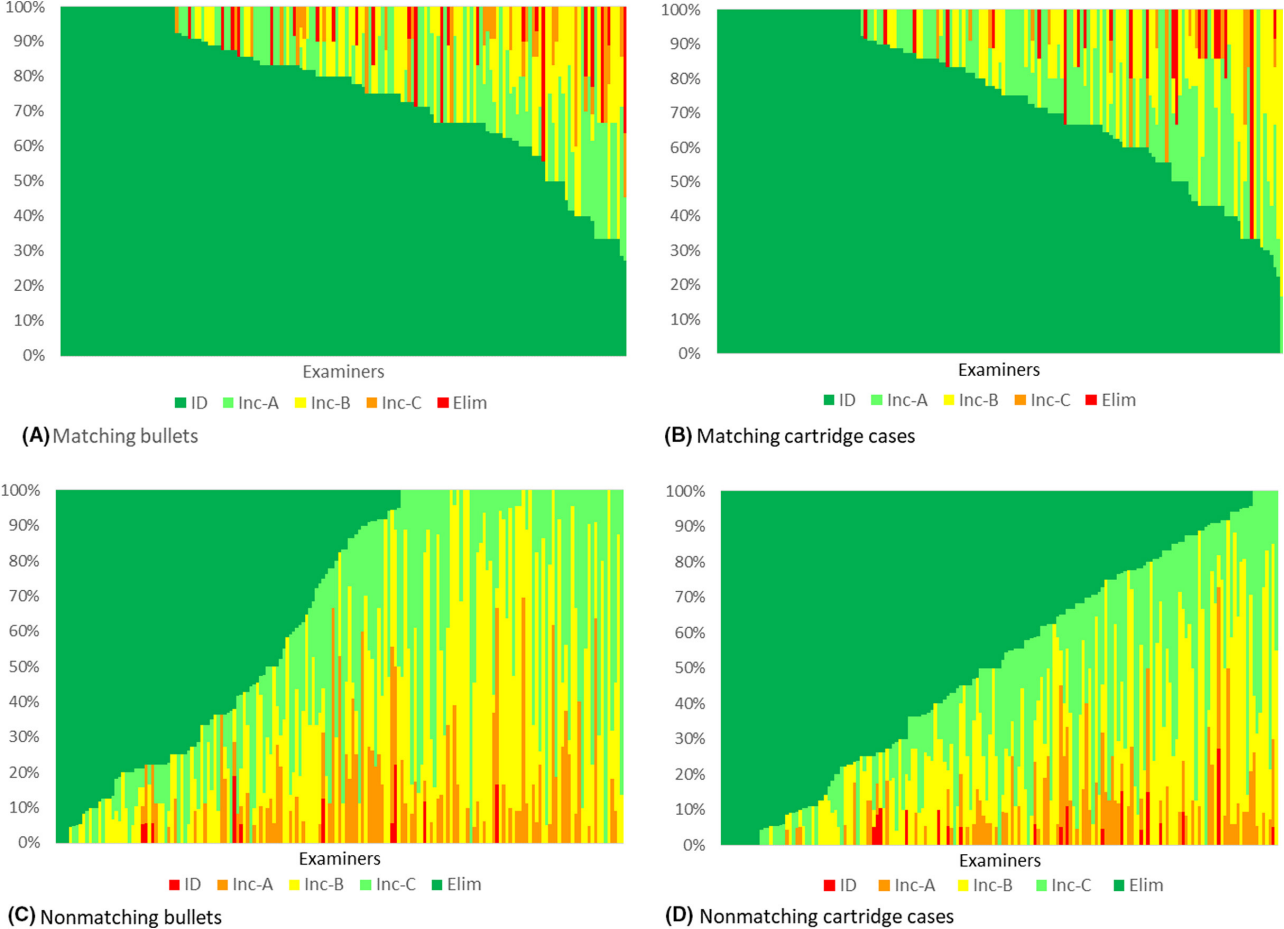
Percentage of completed comparisons (which varies by examiner) in which each AFTE decision category was invoked by each examiner. Decision data were sorted in descending order by correct decision and shown in green, either by ID (for matching sets, panels A and B) or by Elim (for nonmatching sets, panels C and D). [Color figure can be viewed at wileyonlinelibrary.com]

Figure 2 shows a high rate of definitive conclusions by examiners, particularly for known matches. Many examiners correctly identified every set that they compared (i.e., declared not Inconclusives) of known matching cartridge cases (26% of examiners) and bullets (20% of examiners). Figure 2 also illustrates poor performance by a few examiners. The worst performers in each comparison set category declared: 4 of 18 (22%) and 4 of 22 (18%) nonmatching bullet sets to be Identifications; 3 of 11 (27%) and 3 of 20 (15%) of nonmatching cartridge case sets to be Identifications; 4 of 9 (44%) and 4 of 11 (36%) of matching bullet sets to be Eliminations; and 2 of 3 (67%) of matching cartridge case sets to be Eliminations.

**FIGURE 3:**
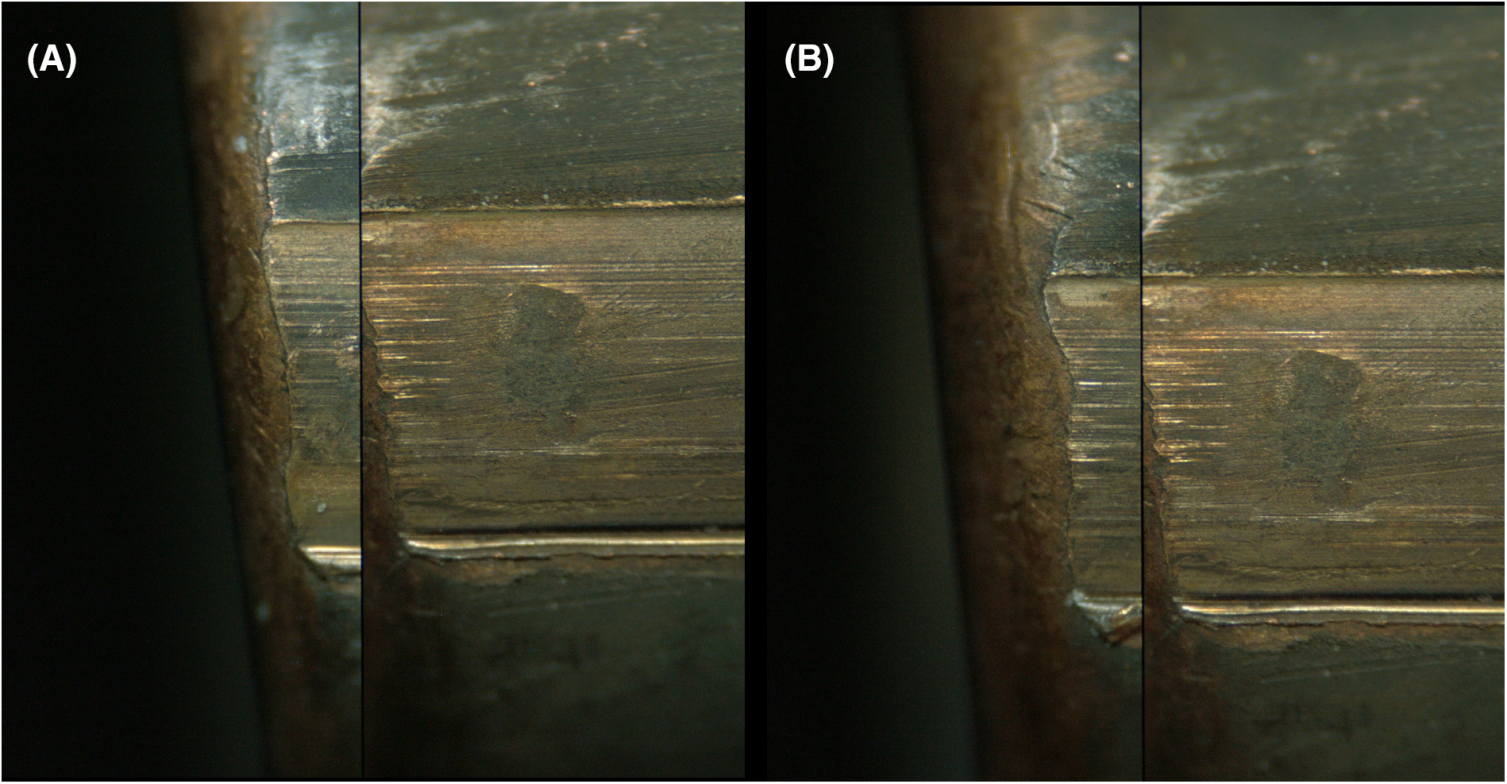
(A) Known nonmatching fired bullet specimen pairs that were incorrectly identified (false positives) and (B) A known‐to‐known comparison from the same sample set is shown for reference. The land impression with the highest degree of similarity is shown. [Color figure can be viewed at wileyonlinelibrary.com]

**FIGURE 4:**
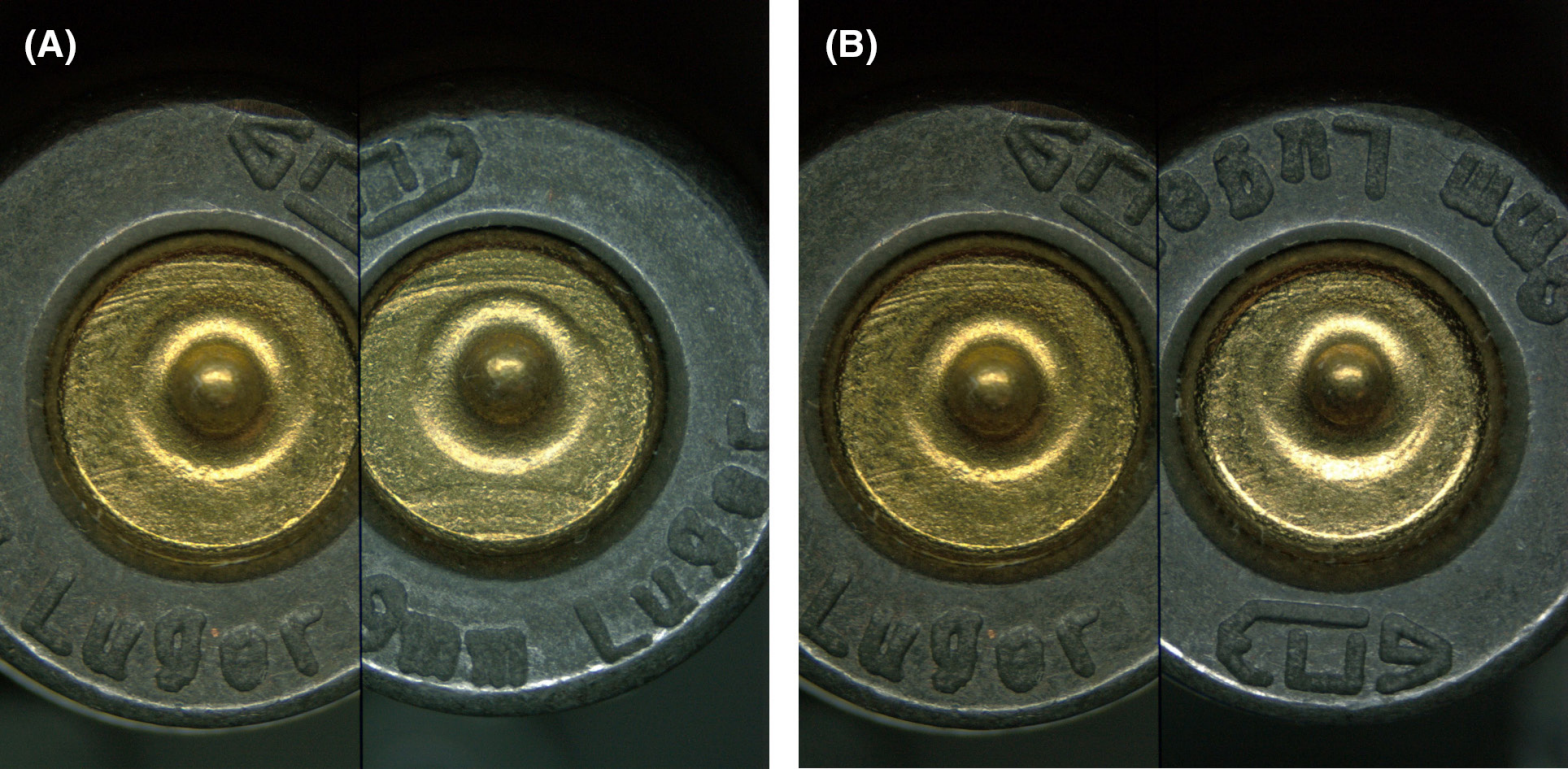
(A) Known nonmatching fired cartridge case specimen pairs that were incorrectly identified (false positives) in a known‐to‐questioned comparison and (B) A known‐to‐known comparison from the same sample set is shown for reference. [Color figure can be viewed at wileyonlinelibrary.com]

**FIGURE 5:**
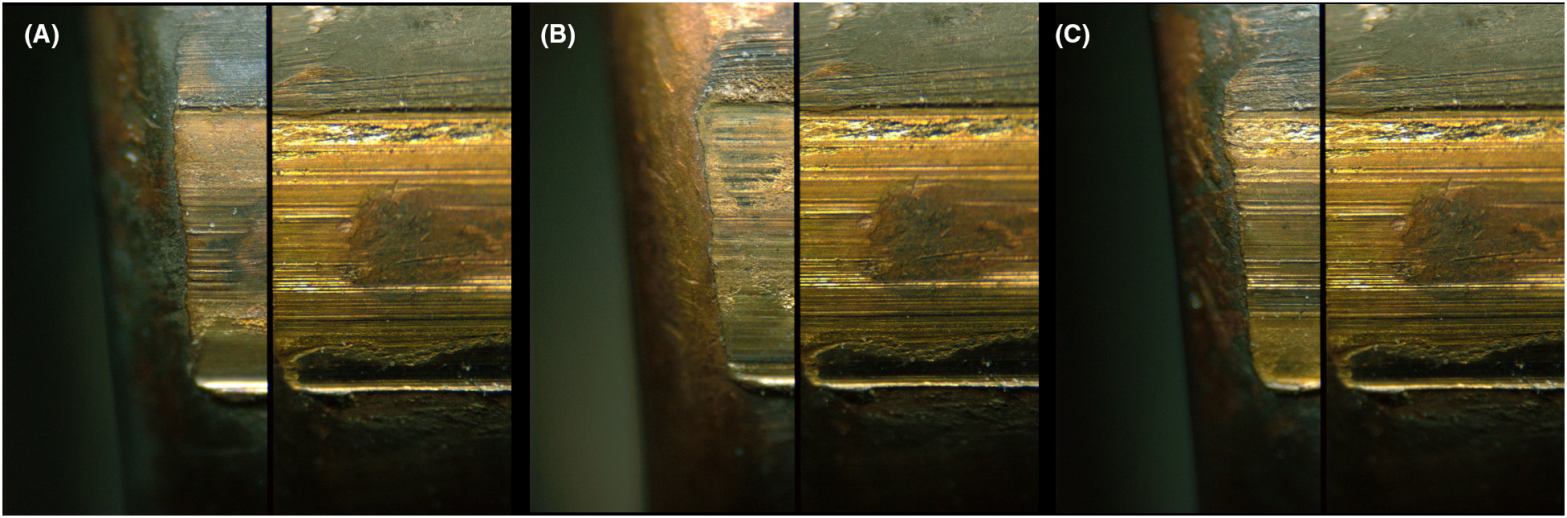
(A) Known matching fired bullet specimen pairs that were incorrectly eliminated (false negatives) in a known‐to‐questioned comparison. (B) Same specimen pair as in panel a, showing a different land impression, and (C) A known‐to‐known comparison from the same sample set is shown for reference. [Color figure can be viewed at wileyonlinelibrary.com]

**FIGURE 6:**
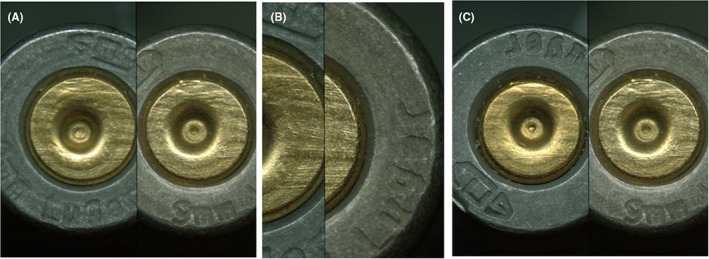
(A) Known matching fired cartridge case specimen pairs that were incorrectly eliminated (false negatives) in a known‐to‐questioned comparison, (B) Detail of panel (A), and (C) A known‐to‐known comparison from the same sample set is shown for reference. [Color figure can be viewed at wileyonlinelibrary.com]

Examples of comparisons that resulted in false‐positive errors are provided in Figures 3 (bullets) and Figure 4 (cartridge cases). Examples of comparisons that resulted in false‐negative errors are provided in Figure 5 (bullets) and Figure 6 (cartridge cases). In addition to exemplifying erroneous conclusions, Figures 3, 4, 5, 6 illustrate the difficulty level of many of the comparisons in this study.

An obvious concern is a possibility that error probabilities are different for individual examiners. If true, then regarding each comparison in the entire collection of examinations of matching bullet sets as having the same probability of being mistakenly labeled an Elimination (for example) is not an appropriate assumption. To examine this possibility, chi‐square tests for independence were performed on tables of counts with 173 rows (one for each examiner) and 5 columns for examination results. For matching sets, the proportions of Identification evaluations versus pooled Elimination and Inconclusive evaluations were compared, and for nonmatching sets, the proportions of Elimination evaluations versus pooled Identification and Inconclusive evaluations were compared. Pooling of counts was used for these statistical tests because errors are relatively rare and, if maintained as a separate category, would result in many zero counts, which are problematic in chi‐square tests, e.g., [48, pp. 156–157]. For both matching and nonmatching sets, and for both bullets and cartridge cases, the hypothesis of independence was rejected (*p* < 0.001) and the effect size was large (Cohen's d > 1) [49, pp., 24‐27], strongly suggesting that the probabilities associated with each conclusion are not the same for each examiner. Consequently, the most common methods of computing confidence intervals for proportions based on an assumption of equal probabilities for each evaluation category, e.g., the Clopper–Pearson intervals [50], are not appropriate.

A more appropriate procedure assumes that each examiner has an individual error probability that these probabilities are adequately represented by a beta distribution, which is a flexible two‐parameter probability distribution on the unit interval, across the population of examiners, and that the number of errors made by each examiner follows a binomial distribution characterized by that examiner's individual probability. Estimates and confidence intervals for the false‐positive error rate were also calculated using a beta‐binomial model, as in the Baldwin study [7]; an example of its use in another application is given in [51]. Usual confidence intervals, in contrast, are based on an assumption that there is only one relevant binomial distribution, and that all examiners operate with the same error probability, an assumption our analysis strongly contradicts. Based on the beta‐binomial model, maximum‐likelihood estimates and 95% confidence intervals for false‐positive and false‐negative error probabilities, integrated over all examiners, were calculated using the R statistics package, including the VGAM package [52, 53]. An expanded explanation is provided in Supplement S2. The results are summarized in Table 5. The maximum‐likelihood estimates and confidence intervals are estimates of the mean of the examiner‐specific error probabilities.

**TABLE 5:** Maximum‐likelihood point estimates and 95% confidence intervals for overall error probabilities, assuming different error probabilities for each examiner

	Point estimate	Lower 95% confidence limit	Upper 95% confidence limit
Bullet comparisons			
False‐positive probability	0.66%	0.31%	1.42%
False‐negative probability	2.87%	1.89%	4.26%
Cartridge case comparisons			
False‐positive probability	0.93%	0.55%	1.57%
False‐negative probability	1.87%	1.16%	2.99%

Table 6 gives the commonly reported indices of sensitivity and specificity computed from our data. Sensitivity is defined as the number of Identification evaluations reported divided by the number of total known matches based on ground truth. It is a measure of the study participants' ability to identify a match between two specimens when they are from the same source. Similarly, specificity is the number of Elimination evaluations reported divided by the number of total nonmatches based on the ground truth. Note that sensitivity would be 1 minus the false‐negative error rate, and specificity would be 1 minus the false‐positive rate, if Inconclusive evaluations were not allowed (and not accommodating variation in individual examiner error rates). These indices are simple ratios of bulk counts, and in light of our discussion concerning unequal error probabilities among examiners, are intended primarily for comparison to other studies rather than as preferred estimates of meaningful underlying parameters.

**TABLE 6:** Computed indices of sensitivity and specificity

	Calculation	Percent
Bullet comparisons		
Sensitivity [identifications]	1076/1405	76.60%
Specificity [eliminations]	961/2842	33.80%
Cartridge case comparisons		
Sensitivity [identifications]	1056/1420	74.40%
Specificity [eliminations]	1375/2835	48.50%

Higher values exist for sensitivity while lower values were obtained for specificity. By comparison, another study of cartridge cases examined using light microscopy reported 80.08% sensitivity and 12.50% specificity [30], while a fingerprint analysis study noted 68% sensitivity and 87% specificity [54]. Lower specificity values for firearms comparisons indicate that it was more difficult for examiners to justify an Elimination decision within ground truth nonmatches than an Identification within ground truth matching specimens. This observation accords with research in cognitive science, which has demonstrated that the more similar are two images, the more difficult it is to say that they are different, particularly as it becomes harder to bring the images into alignment [55]. Also contributing to lower specificity is the fact that 7% of examiners were habituated to laboratory policy not to declare Elimination based on individual characteristics. In view of the difficulty of the comparisons encountered in this study, lower specificity, i.e., reticence in making decisions of being “definitely different” (i.e., Elimination), is not unexpected.

Anonymized results of all comparisons of bullet and cartridge case sets that were conducted by the participating examiners are provided in Supplement S3.

## 4 DISCUSSION

### 4.1 Accuracy

The error rates in this study are somewhat higher but generally consistent with the overall error rates reported in recent firearms studies with an open set design (Table 7) [4, 12, 14, 15, 16, 17, 56]. Exact correspondence in error rates across different studies would not necessarily be expected due to differences in study design, in the firearms and ammunition chosen to produce test specimens, and in the way that error rates are calculated. For cartridge cases, the false‐positive error rates in this study are comparable to the overall error rates reported by Baldwin et al. [4]. Estimated error rates are higher in this study than those reported in a recent study involving bullets fired from 30 consecutively machined Beretta barrels, which reported false‐positive rates of 0.08% (with 95% upper confidence limit of 0.4%) and false‐negative error rates of 0.16% [14, 57].

**TABLE 7:** Recent open set decision analysis studies and reported error rates of firearms examiners

Author(s)	Firearms			Examiners	Comparisons		Error rate (FP/FN), percent	
	No.	Manufacturer	Seq.^b^	No.	Cartr. Cases	Bullets	Cartr. Cases	Bullets
Baldwin et al., (2014)	25	Ruger	R	218	3270		0.94^a^/0.37^a^	
Kerkhoff et al., (2015)	10	Various	R	11	341	55	0/0	0/0
T. Smith et al., (2016)	8	Various	R	31	693	995	0.14/0.43	0/0.10
Kerkhoff et al., (2018)	1 39	Sig Sauer Glock	R R	10	344		0/0	
J. Smith (2020, 2022)	35	Various	C	74		7420		0.08^a^/0.16^a^
Law & Morris (2021)	20	Various	R	17	340		0.28/0	
Best et al., (2022)	10	Thompson/Center Arms	C	44		660		0.46/1.82
This Study (2022)	23 4 10 10	Beretta Beretta Ruger Jimenez	C R C C	173	10,110	10,020	0.93^a^/1.87^a^	0.66^a^/2.87^a^

^a^ Maximum likelihood estimate.

^b^ Seq: C/R (Consecutive/Random manufacturing sequence).

Experimental parameters of the present study were challenging by design, including using particular firearms that tend to mark more poorly, steel versus brass cartridge cases, steel‐jacketed bullets, and promoting the presence of subclass characteristics [33]. The consecutively or sequentially manufactured barrels and slides used in this study suggest a source of subclass characteristics. Anecdotally, the Jimenez firearm is known to generate gross marks with high occurrences of subclass characteristics both for breech face marks and firing pin impressions [58], as compared to higher cost‐point firearms such as the Beretta. The lack of a tilting barrel recoil mechanism in all the firearms used in this study increases the difficulty level of comparisons due to the absence of distinctive aperture shear marks. Higher false negatives recorded are possibly also due to greater difficulties when faced with the steel Wolf Polyformance cartridge cases rather than softer brass used in other studies. Many examiners commented that they felt brass provides better marks for Identification than steel. Lacking access to the firearm that produced the known specimens, as is typical in most casework, also made comparisons more difficult.

Several examiners commented that, without having the actual firearm in hand to test, they found it difficult to render an exclusion, particularly when there was no information given as to the firing sequence gap between the collection of the unknown and the collection of the known exemplars. This limitation is elaborated on in a proposed standard [59]. Casework comparisons often offer the opportunity to produce test fires within a relatively close interval from the shooting incident under investigation. (However, studies have shown little effect on Identification performance in the absence of the firearm [4, 10, 11, 39].)

Errors tended to be concentrated within a relatively small number of examiners (Table 4, Figure 2), as observed in other studies [4, 14, 15]. Examination of the data using chi‐square tests for independence showed that the cited error estimates cannot be applied equally to all examiners. Most examiners will perform better than the point estimates in Table 5, while a few will perform more poorly. No errors were made by approximately 80% of examiners (Table 4). Point estimates and confidence intervals were calculated under the assumption (supported by our analysis) that examiners have different error probabilities and that the collection of examiner‐specific probabilities can be represented by a beta distribution.

The confidence intervals shown in Table 5 should not be interpreted as bounding the error probabilities of any one examiner. Again, the error probabilities of individual examiners are assumed to be different, and the data available for any one examiner are limited. A valid alternative explanation of the confidence interval is that, if many examiners were randomly selected from the population and individually asked to make a single determination for a (different) comparison set, the intervals specified would bound, with stated confidence, the overall proportion of errors made in this process. It should also be noted that this method is not completely assumption free (even though the assumptions are less restrictive than those on which the Clopper–Pearson intervals are based). Specifically, it is assumed without formal evidence that the beta distribution is appropriate for modeling the population of examiner‐specific error probabilities. The flexibility of the beta‐distribution family (i.e., the variety of shapes the distribution can take, controlled by its parameters) ensures that the methodology can be appropriate for a wide variety of situations. Because the examiner‐specific error probabilities are not directly observable, and there is relatively limited information available on the accuracy of each examiner's determinations, it would be difficult to build a supportable case for more appropriate distribution. Even if a different distribution was available, the beta distribution is certainly a more appropriate approximation than the single‐value distribution assumed by the Clopper–Pearson approach.

Given the above provisos, the results in Table 5 should still be considered approximate since the model of a firearm and the positioning of known and questioned specimens in the firing sequence for a firearm also appear to affect error probabilities, and these considerations are not taken into account in this calculation; these effects will be discussed in another paper. Still, differences among examiners are likely the greatest source of nonindependence in the data, and the assumptions underlying the method used here are more appropriate than those upon which simpler methods are based.

The participants in this study were directed to use the AFTE range of conclusions [46], which predominates in North America, to express their comparison decisions. Alternative scales, which describe conclusions in terms of strength of support, are under consideration [59, 60]. If adopted by the community, the value of studies using the AFTE range will endure. The proposed scale is highly comparable to the AFTE range, being essentially a change in nomenclature. The term Elimination is replaced by Exclusion, while Identification remains. The middle three conclusions of the proposed scale closely approximate the definitions of the three AFTE levels of Inconclusive.

### 4.2 Inconclusive decisions

Forensic firearms comparison must be regarded as at least a two‐level process. The first level is an evaluation of the class characteristics. If they are congruent, the second step involves comparing the quality and quantity of microscopic correspondence of individual characteristics. (Individual laboratory policy may permit elimination using a difference in microscopic marks at the second step.) As with any instrument (the examiner being the instrument), there are limits on their ability to the interpretation of the quality/quantity of the data/information presented. For many reasons, fired bullets and cartridge cases do not always carry marks sufficient to support a definitive conclusion of Identification or Elimination [46, 59, 61]. Sufficient agreement in quality and/or quantity of individual characteristics is dependent on toolmark reproduction and/or survivability. The following factors may influence toolmark reproduction (some apply only to casework specimens):

- Limited obturation—obturation is the enlargement of a cartridge case or a bullet base to seal the chamber during the expansion of gases. When there is incomplete/limited obturation, the reproduction of the toolmark is negatively affected. Factors such as ridged substrates and/or loose manufacturing tolerances can impact the reproduction of a toolmark.
- Intermediate substrate—whether intentional (e.g., primer lacquer) or accidental, an intermediate substrate such as debris (e.g., lubricant, dirt, and sooting) can inhibit toolmark reproduction.
- Interference—secondary toolmark obstructs primary toolmark comparison, e.g., cartridge case mouth striations on a bullet merging with striations produced from the barrel.
- Longevity of toolmark (persistence)—through long‐term use of a tool, erosion of the original toolmark can occur.
- High velocities—when velocities are high, the increased pressure on the bearing surface of a bullet can reduce toolmark reproduction.
- Intentional alteration—numerous methods to obliterate an original toolmark through mechanical means exist (e.g., sanding and grinding) to conceal the originally manufactured toolmark; however, this generates a new toolmark that is different from the original.
- Environmental exposure—depending on the environmental conditions and/or the metal substrate, the original toolmark is susceptible to alteration due to corrosion.
- Damage—due to the velocity of an impact or the active nature of a crime scene (e.g., evidence being trodden upon), toolmarks on bullets/cartridge cases can be damaged, obscured, or obliterated.
- Substrate—may not be suitable for toolmark reproduction (e.g., hard metallics)

When there is inadequate reproduction of a toolmark, the quality and/or quantity of individual characteristics available for comparison may be insufficient to conclude an Identification or Elimination. The forensic community, as well as independent researchers, are in agreement that the appropriate recourse for an examiner is then a decision of Inconclusive [62, 63, 64, 65, 66, 67]. By recording an Inconclusive decision, an examiner is providing a conclusion that the information/data observed do not meet the high standards for Identification or Elimination. Rather than guessing, they say they are unsure and return an Inconclusive decision. Biederman et al. [63, 68] make trenchant arguments for the utility of Inconclusive decisions and that they should not be considered errors. First, they point out that calling an Inconclusive conclusion from a known match comparison an error is a contradiction in terms, being that an Inconclusive decision makes no reference to ground truth. Second, they show through formal decision theoretic analysis that an Inconclusive decision actually has high practical value (utility), particularly in individual cases and within the precept that false positives are highly undesirable. In this study, we have not imputed error to Inconclusive conclusions.

In a controlled research study where the ground truth match/nonmatch status of every comparison is known, one may readily count the number of known matches and known nonmatches that were deemed Inconclusive by examiners, as in Table 2. Such counts have been dubbed “incorrect Inconclusives,” which some authors propose should be considered systematic errors inherent to the process of firearms comparisons, as they fail to indicate what is known, although emphasizing that they should not be considered individual errors [15, 65, 67, 69]. Such counts are highly dependent on the overall difficulty level of comparisons in each study and on intra‐ and inter‐examiner variations in decision‐making [1, 70].

Another approach that has been advocated for casework (where true match status is unknowable) is to convene a group of experts to arrive at a consensus opinion. Any deviation from consensus would be considered an “incorrect Inconclusive” [67, 69]. The general approach to characterize so‐called “incorrect Inconclusives” would be to have a group of examiners each compare the same group of specimen pairs to establish a consensus opinion. The consensus method can also be applied to a research study. One such study, involving 13 firearms examiners, achieved unanimity only for the 7 known matches among 20 comparison sets, but demonstrated a somewhat higher proportion of ground truth conclusions for most examiners by including some of their Inconclusive decisions that fell within the interquartile range of the consensus [15]. Wide variation in ground truth accuracy for each set was attributed to an inadequate number of examiners forming the consensus group. A study assessing the value for Identification of latent fingerprints by 23 participants achieved unanimity on 48% of 520 mated pairs [1]. Another study involving the analysis of 12,279 palm prints by 226 examiners found a high level of variability in decisions of value, with 25% disagreement from consensus [71]. The latter study also found the unnerving result with respect to casework that, in 45 instances, an Identification conclusion differed from the consensus decision of exclusion (reached by a large number of examiners), but the consensus was wrong in 36 of those instances.

Seeking consensus was first suggested to support an accused's right to appeal [67] or to assess whether particular latent fingerprint examiners within a laboratory may be overly cautious or aggressive—but “not necessarily wrong in absolute terms” [72, section 3.3.6.2]. (Technical review of laboratory reports prior to release, integral to many quality‐assurance protocols, is a related concept that usually involves two experts, while consensus involves a group.) Counting known matching and known nonmatching sets that were judged Inconclusive (at any subdivision) would reveal examiners who treat some comparisons that offer minimal, inadequate, or ambiguous discriminating information either more conservatively or aggressively than other examiners but might also imply they made errors. Several practical limitations beset the method of consensus, including operational overhead, determining membership and size of the august group, use of majority vs. unanimous opinion, and differences in outcome arising from differences in group membership, training, and level of conservatism.

In reporting the results of the present black box study of examiner accuracy, which is intrinsically agnostic to process, we have taken the position that Inconclusive conclusions shall not be considered errors. Combining the false positive error rate with the rate of Inconclusive decisions would exemplify the same ubiquitous “systematic error” due to limitations in our ability to sense or measure any natural phenomenon. It would result in an inappropriate and misleading amalgam of an important metric (false‐positive rate) with one that is noninculpatory and inherently agnostic (Inconclusive rate) [1, 70]. Inconclusive decisions are not systematic errors; rather, they are an essential part of the firearms discipline and Inconclusive decisions provide a check against bad Identifications.

### 4.3 Bias mitigation

A reliable research study is designed to anticipate, recognize, and mitigate potential sources of bias [33, 41]. Some initial volunteers discontinued their participation in the project without reviewing any specimen packets once they realized that their daily workload was incompatible with the high level of effort required to participate. Research can rarely test an entire population so must address the representativeness of a population sample. We solicited volunteers among American firearms examiners representing every employment situation except self‐employment. Thus, it was not a random sample, but a sample of convenience.As volunteers, the participants were, of necessity, self‐selected and aware of study participation due to statutory requirements for the protection of human subjects in research (Materials and Methods section) and by being willing and able to devote extensive time and effort above and beyond the demands of casework. Selection bias is a potential concern that forms the basis for assertions that self‐selection/voluntary participation results in a no‐representative sample (the participants) from the sampling frame (all forensic examiners). The implied consequences of nonrepresentativeness are that the participants self‐select into the study for reasons that might cause them to perform better than the examiner population at large, lowering the calculated error rate. There is no empirical basis for an assumption of superior performance by those who opted for participation. Our assumption in designing the experiment is that any potential sources of bias are compensating, and thus, the sample pool is sufficiently representative of the larger population.

We specifically requested that voluntary participants be “qualified examiners,” in the assumption that most crime laboratories do not classify their qualified examiners into various arbitrary and subjective performance or proficiency levels. An examiner is either “qualified” or not; “proficient” or not, without further gradation.

Another source of potential bias is the Hawthorne effect, which is a phenomenon in which individuals behave differently when they are being observed [73]. Similar effects have been postulated related to selection bias, viz., that individuals who engage diligently for the duration of the study are somehow more skilled as examiners, while those who terminate their role in the research prior to completion are less skillful or less conscientious. The resulting imputation is that error rates are artificially suppressed. In the case of our research, compensating phenomena offset potential Hawthorne effects. Increased “diligence” in performing the comparisons for the experiment that might result in “better” results should be offset by the absence of typical casework protocols that would reduce errors further, such as secondary reviews and blind verifications. Furthermore, we could reasonably postulate diminished “diligence” since the experimental specimens do not represent “real” casework, with all the attendant consequences.

As noted above, some enrolled examiners did not complete the full course of comparisons. Among the examiners making false‐positive Identifications of either bullets or cartridge cases, their degree of participation in processing multiple submissions of sample sets was similar to that observed among examiners making no false Identifications. To test for substructure in the sample population due to unequal participation, results were stratified according to what was reported by two distinct groups of examiners: those who performed 345 bullet comparisons and those who performed 690 comparisons (Table 8). Because the reported number of errors is small, being equal to 0 in one case, a two‐sided Fisher's exact test was used to test for nonrandom associations between results in the two subgroups. The exact probability is 0.309, indicating no significant difference between the accuracy of examiners who withdrew from the study and those who remained.

**TABLE 8:** Errors reported by two subgroups of examiners, those who performed 345 bullet comparisons and those who performed 690 comparisons

Bullet comparisons				
Subgroup	Examiners	Comparisons	False positives	False negatives
A	59	345	0	6
B	113	690	10	27

Nor were there any indications of population substructure due to the use of the CMS method (which involves determining whether the number of consecutive matching striae meets a minimum criterion for correspondence that was empirically determined from best known nonmatches [36, 74]) or whether the employing laboratory was accredited. Among 10 examiners who committed false‐positive errors with bullets (Tables 3, 7 and Figure 1), one included CMS as part of the comparison process (a very small fraction of all examiners did so; the number is withheld to protect anonymity), and another is employed by a nonaccredited laboratory (of the 18 participating examiners in nonaccredited laboratories). Similarly, for the 18 examiners making false‐positive Identifications of cartridge cases (Table 4 and Figure 1), one (the same one) is a practitioner of the CMS approach for bullets and another is employed by a nonaccredited laboratory.

## 5 CONCLUSIONS

This black box study demonstrated a high level of performance by 173 qualified firearms examiners who performed 8640 challenging comparisons. No false‐positive or false‐negative errors were made by the majority of examiners when examining bullets or cartridge cases (80% and 79% of examiners, respectively). Estimates for overall false‐positive and false‐negative error probabilities were calculated as 0.656% and 2.87% for bullets and 0.93% and 1.87%, for cartridge cases, respectively. The 95% confidence intervals for false positives and false negatives are (0305%, 1.42%) and (1.89%, 4.26%), respectively, for bullets. Similarly, for cartridge cases the 95% confidence intervals are (0.548%, 1.57%) and (1.16%, 2.99%) for false positives and false negatives, respectively.

This study presented a challenging test of examiner capabilities by using: a fully randomized open set design; firearms that do not produce aperture shear/firing pin drag; conditions promoting the production of subclass characteristics (via separation in manufacturing sequence and/or related to aspects of firearm design); steel ammunition (cartridge cases and jacketed bullets); variable separation in the firing order (up to 850 firings); no verification by a second examiner; and unavailability of firearms or barrel casts, either for further analysis or production of additional reference specimens.

The majority of errors were made by a limited number of examiners. For example, 13 examiners account for almost half of all the errors (54 of 112). A subset of these 13 is the 6 most error‐prone examiners, who accounted for almost 30% of the total errors (33 of 112). Because error rates vary by the examiner, 95% confidence limits on error probabilities were estimated using a beta‐binomial model that assumes a separate error probability for each examiner.

The population of participating examiners was a sample of convenience, but there are no discernible indications of any characteristics that might set those examiners apart, including duration of study participation, laboratory accreditation, or use of CMS. Conclusions are somewhat tenuous because the fraction of participating examiners who made errors is small. The study sample likely was reasonably representative of the population of qualified firearms examiners who are employed by public forensic laboratories in the United States.

Examiners demonstrated a high rate of definitive conclusions, particularly for known matches. Differences in the observed rate of definitive conclusions are attributable to the challenges presented by the particular comparison sets each examiner received, as well as to examiner skill and risk tolerance. For ground truth matches, the overall rate of Identification was similar for bullet and cartridge case sets, while the rate of Inconclusive decisions was somewhat higher for the latter. For ground truth nonmatches, relative to comparisons of cartridge case sets, bullet sets resulted in fewer Eliminations, and consequently more Inconclusive decisions.

The results of this study add to the ever increasing body of empirical data that firearms examiners conduct comparisons with a high level of accuracy. This and related studies address the “known or potential rate of error” of the Daubert court [75], Recommendation 3 of the NAS Report [2], and several recommendations of the PCAST report [3] by measuring the accuracy and reliability of forensic analyses. Black box studies assess the overall reliability of a forensic discipline, not that of any particular examiner. The present study included a reasonably large and representative sample of practicing examiners, and many comparisons in a range of difficulties, conducted in a declared double‐blind, open‐set format. The results will offer additional resources to the courts as they weigh the admissibility and value of firearms testimony [66, 76].

## CONFLICT OF INTEREST

The authors have no conflicts of interest to declare.

## Supporting information


Appendix S1

Appendix S2

Appendix S3
