# Supplementary material for: Accuracy of comparison decisions by forensic firearms examiners
Source: J Forensic Sci. 2022 Oct 1;68(1):86–100. doi: 10.1111/1556-4029.15152 (PMC10092368; doi:10.1111/1556-4029.15152)
Supplement: Supplementary file 1 — Appendix S1 [file JFO-68-86-s001.docx]

**SUPPLEMENT 1 Bar Code Labeling, Distribution, and Tracking of Specimens**

Two-dimensional bar codes were used to track all bullets and cartridge cases, as shown in Figure S1. As the study planned to assess repeatability as well as accuracy, these codes made it difficult for participants to identify previously viewed specimens. Specimens (both cartridge cases and bullets) were labeled as Ks to designate known specimens and labels that had no extra letter were used for questioned (Q) specimens. This was done so that if an examiner mixed up the known and questioned specimens in a comparison set during analysis, they could still be distinguished. Bullets were epoxied on plastic mounts to facilitate handling and provide a place for affixing labels (Figure S1b). Cartridge case labels were placed on areas where minimal marks were present. After all the specimens were labeled, they were inventoried by barcode labels, which were linked to additional information associated with the specimen: serial number of the firearm, test firing-order range, specimen type, and whether the specimen was a questioned or known specimen. This information was used to track specimens, verify the "ground truth" for all test packets assembled, and for those repackaged for subsequent re-examination in the assessment of examiner reliability and reproducibility.


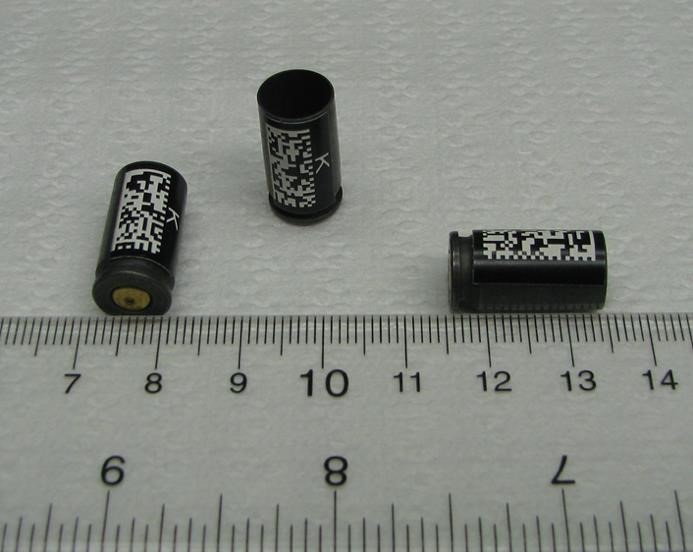

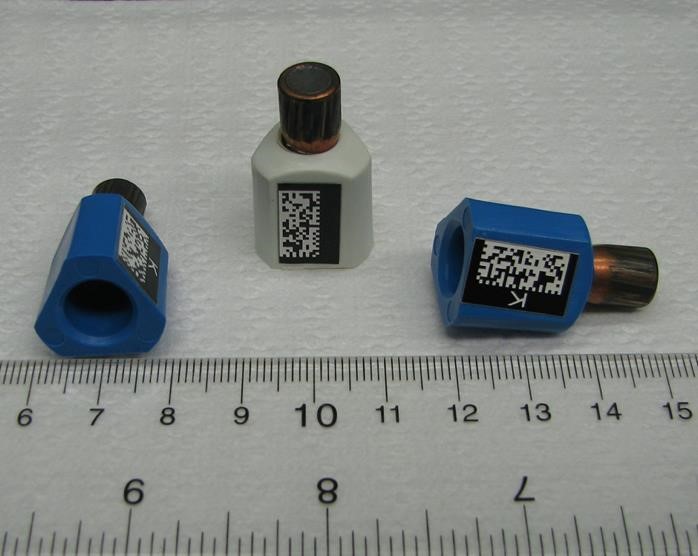


a. b.

FIGURE S1 Specimens for examination. a) Cartridge cases showing 2D bar code. b) Bullets showing plastic mounts and 2D bar codes

The test packets were distributed through a series of mailings, with examiners being asked to complete as many mailings as possible (up to a total of six) as their time and workload allowed. Each test packet assembled for mailing consisted of the 15 bullet and 15 cartridge case comparison sets to be analyzed, an instruction sheet, and answer sheets [1]. A 3-page survey form was included in the first mailing to obtain demographic and laboratory procedure information [1], and summarized survey information will be presented in an upcoming publication. Each test packet was assigned a primary numeric code to track the comparison sets. The specimen set pairings within a given packet were maintained throughout the study.

Items for each mailing were placed into a Tyvek envelope, sealed, and labeled with a secondary identifying code linked to the unique primary identifying group number for a given test packet. The secondary code was a 3-digit alphanumeric identifier – the first two letters designated the participant while the third letter or number indicated the mailing. This secondary code is what enabled packets to be shared between the experimental group and the communication group; the primary identifiers were not made known to the communication group. A return Tyvek envelope, also labeled with the secondary-identifier code, was included in the assembled packet and used by examiners to return the specimens and their analysis results sheets. Figure S2 shows the contents of a typical first mailing.


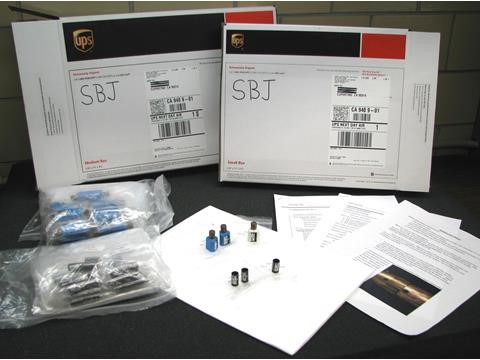


FIGURE S2 Specimen packet components, including the bullet and cartridge case test sets, forms, and shipping and return boxes

The sealed Tyvek envelope was transferred to the communication group, who placed the envelope in a shipping box and mailed it to the participating examiner, whose identity was known only to the communications group. Returned packets were logged and inspected upon arrival by the communication group for any examiner-specific identifying information, prior to transferring the sealed Tyvek bag containing the analysis results to the experimental/analysis group for scoring, database entry, and verification of the results.

If a decision error was noted, the comparison set was barcode-read and the information compared to the known “ground truth” to verify the error. Analyzed test packets needed for the assessment of repeatability and reproducibility were repackaged before redistribution. Each specimen in each comparison set was visually examined and gently cleaned of any debris or marks and assigned new randomized set and group numbers. The repackaged test packet was barcode-read once again, to verify the “ground-truth” of the reassembled packet, prior to use in succeeding mailings.

**References**

1. Monson KL, Smith ES, Bajic SJ. Planning, design and logistics of a decision analysis study: The FBI/Ames study involving forensic firearms examiners. Forensic Sci Int Synergy. 2022;4:100221. doi: 10.1016/j.fsisyn.2022.100221.
