# Supplementary material for: Accuracy of comparison decisions by forensic firearms examiners
Source: J Forensic Sci. 2022 Oct 1;68(1):86–100. doi: 10.1111/1556-4029.15152 (PMC10092368; doi:10.1111/1556-4029.15152)
Supplement: Supplementary file 2 — Appendix S2 [file JFO-68-86-s002.docx]

## SUPPLEMENT 2 Error Probability Confidence Intervals

###

### Point estimation and confidence interval for false positive errors

For specificity, this discussion will address the estimation of the probability of false positive conclusions. As noted in the text, this is regarded as the probability that a true non-match is classified as an Identification.

The simplest, and probably most intuitive statistic that might be considered to estimate this probability is the number of false Identification conclusions divided by the total number of the six possibilities in the AFTE Range of Conclusions (Identification, Inconclusive-A, Inconclusive-B, Inconclusive-C, Elimination, or Unsuitable) [1], from among those comparison sets that are non-matching, i.e., the proportion of errors made over all examiners and non-matching comparison sets. Because this ratio is a simple proportion, it is then tempting to use a standard statistical method for computing a confidence interval based on an assumption that all examiner conclusions are independent and have the same error probability; the Clopper-Pearson [2] method is perhaps the most often-used. As strongly suggested by the analysis presented in the main text, the assumption that the same error probabilities apply to each examiner is suspect. As a result, the ``simple estimate’’ described above is actually an estimate of a composite error probability arbitrarily weighted toward the characteristics of examiners who evaluated more comparison sets, and any confidence interval methodology that regards all errors as equally probable is based on an inappropriate mathematical model and so should not be trusted.

In place of this, the confidence intervals employed in this analysis are based on methodology that allows for different error probabilities for each examiner. This approach is based on two different families of probability distributions:

1. **Beta distribution**: The beta distribution is a continuous probability distribution over the interval [0,1], which has a flexible shape that is governed by two parameters, e.g., [3]. The analysis regards each examiner’s error probability as an independent ``draw’’ from this distribution, allowing them to be different. The analysis does not require that these (true) error probabilities are actually observed, but incorporates indirect information based on the number of errors made by each examiner.
2. **Binomial distribution**: The binomial distribution is a discrete probability distribution over non-negative integer values up to a specified value, often called *n*, one of the parameters of the distribution, e.g., [3]. The other parameter is a probability, and the modeled (random) variable is the number of errors that are made if each of *n* independent conclusions is subject to this same probability of error. The binomial distribution (alone) is the basis of the most commonly used confidence intervals associated with proportions. In the method used in the analyses presented here, the number of errors made by each examiner is modeled as a ``draw’’ from an individual binomial distribution characterized by the number of comparison sets examined by that examiner, and that examiner’s specific and unknown error probability.

The beta distribution has a mathematical form that is often called *conjugate*, with respect to the binomial. In essence, this means that the two distributions can be combined to form a new distribution appropriate for modeling the expected number of errors to be made by an *unspecified* examiner; that is, a distribution that models the number of errors made by an examiner drawn randomly from among the relevant population of examiners. This property allows for the construction of what statisticians call a *likelihood function –* the mathematical form central to computing point estimates and confidence intervals for the parameters of the beta distribution, based on the numbers of errors made by each examiner (which must be kept separate in the analysis to account for the fact that the error probabilities are not the same for each). The beta distribution parameters, in turn, characterize the distribution of ``true’’ examiner-specific error probabilities. *The maximum likelihood estimates and confidence intervals cited in the main text are estimates of the mean of the examiner-specific error probabilities.*

Note that, given this situation, the confidence interval should not be interpreted as bounding the error probability of *any one examiner*. Again, it is not assumed that these probabilities are the same, and the data available for any one examiner are quite limited. A valid, if artificial, alternative explanation of the reported confidence interval is the following: If many examiners are randomly selected from the population and each asked to make a single decision for a (different) comparison set known to be a nonmatch, the intervals given for false positive probabilities bound, with stated confidence, the overall proportion of errors made in this process.

It should also be noted that this method is not completely assumption-free (even though the assumptions are weaker than those on which the Clopper-Pearson intervals are based). Specifically, it is assumed without formal evidence that the beta distribution is appropriate for modeling the population of examiner-specific error probabilities. The flexibility of the beta distribution family (i.e., the variety of shapes the distribution can take, controlled by its parameters) ensures that the methodology can be appropriate for a wide variety of situations. Because the examiner-specific error probabilities are not directly observable, and there is relatively limited information available on the accuracy of each examiner’s conclusions, it would be difficult to build a convincing case for a more appropriate distribution. (And even if a different distribution really should be used, the beta distribution is certainly a more appropriate approximation than the single-value distribution assumed by the Clopper-Pearson approach.)

###

### Calculation of likelihood-based point estimates and confidence intervals

As noted in the text, the VGAM package [4] was used to compute likelihood-based estimates. As should be expected from the discussion above, the data required for this calculation are the number of examinations made and (of these) the number of errors made *for each examiner individually*, i.e., not combined. (Further information on VGAM is available at the reference cited below.) Given the maximum likelihood estimates of the parameters, calculation of the confidence interval was accomplished via evaluation of the likelihood function over a grid of the parameter values.

1) Maximum likelihood estimates: Estimation of the beta parameters requires data on the number of examinations and errors made by each examiner. Given a vector *y* of error counts and a vector *n* of examination counts (each of length 173 for data taken from round 1), the specific commands used (for false positive case evaluations) and resulting output are:

fit <- vglm(cbind(y,n-y) ~ 1, betabinomial, irho=.9)

coef(fit)

mu rho

0.00933045 0.05026947

These are the maximum likelihood estimates for the two parameters that characterize the distribution; the first (“mu”) is the mean of the beta distribution being used to model the examiner-specific error probabilities, and is the quantity of interest here.

2) Confidence intervals for mu*:* While fitting maximum likelihood estimates for this model is a bit tricky (and so accomplished with the program VGAM [4]), the likelihood function itself is relatively easy to calculate using standard R commands. Specifically, the command:

ll <- 0

for(i in 1:173){

ll <- ll + dbetabinom(y[i], n[i], mu, rho, log = TRUE)

}

can be used to compute the beta-binomial log-likelihood (ll) for specified parameter values mu and rho. (The likelihood function is computed on a log scale here because this is the quantity on which the confidence interval is based.) Because rho is a ``nuisance parameter’’ in this application, the profile-log-likelihood – i.e. for a given value of mu, the maximum of the log-likelihood function over values of rho – is the basis for a confidence interval for mu alone. The log-likelihood function was calculated over a grid of both parameter values, and this was reduced to a one-dimensional grid (over mu) by identifying the mu-specific value of rho for which the log-likelihood is maximized. Using large-sample likelihood theory, the upper and lower 95% confidence limits for mu were identified as the two values for which the profile-log-likelihood is 3.8410/2 = 1.9205 less than the log-likelihood value at the maximum likelihood estimates. (The value of 3.8410 is the 95^th^ quantile of the chi-square distribution with one degree of freedom.)

A more detailed description of the construction of confidence intervals using profile likelihood can be found, for example, in Cox and Snell [5].

### **References**

1. Association of Firearms and Toolmarks Examiners. AFTE range of conclusions. Association of Firearms and Toolmarks Examiners. 2020. https://afte.org/about-us/what-is-afte/afte-range-of-conclusions. Accessed 13 Sep 2022.

2. Clopper CJ, Pearson ES. The use of confidence or fiducial limits illustrated in the case of the binomial. Biometrika. 1934;26(4):404–13.

3. Evans M, Hastings N, Peacock B. Statistical distributions, 3rd edn. New York, NY: John Wiley and Sons; 2000.

4. Yee TW. The VGAM package for categorical data analysis. J Stat Softw. 2010;32(10):1–34. doi: 10.18637/jss.v032.i10.

5. Cox DR, Snell EJ. Analysis of binary data. London, U.K.: Chapman & Hall/CRC Press; 2018.
